# Acid-Catalyzed Esterification of Macauba Pulp (*Acrocomia aculeata*) and Brazil Nut (*Bertholletia excelsa*) Oils of High Acid Values over Mesoporous Silica Functionalized with Propyl Sulfonic Acid

**DOI:** 10.1021/acsomega.5c01099

**Published:** 2025-03-24

**Authors:** José Sebastião C. Vieira, Anderson F. S. Moreira, Ricardo D. Anjos, Ana Lúcia de Lima, Elizabeth R. Lachter, Germildo G. Muchave, Claudio J. A. Mota

**Affiliations:** 1 Escola de Química, Universidade Federal do Rio de Janeiro, Rio de Janeiro. Av Athos da Silveira Ramos, 149, CT, Bloco E, Rio de Janeiro 21941-909, Brazil; 2 Departamento de Desenvolvimento de Ensino, Instituto Federal do Maranhão − Campus Zé Doca, Zé Doca, MA 65365-000, Brazil; 3 Instituto Federal do Maranhão, Campus São José de Ribamar, Rodovia MA 201. S/N-Vila Piçarreira, São José de Ribamar, MA 65110-000, Brazil; 4 Instituto de Química, Universidade Federal do Rio de Janeiro, Rio de Janeiro. Av Athos da Silveira Ramos, 149, CeT, Bloco A, Rio de Janeiro 21941-909, Brazil; 5 WUTIVI University (UniTiva), Av. Da Namaacha, n°188, Belo − Horizonte, Boane, Maputo 1415, Mozambique; 6 INCT Energia e Ambiente, UFRJ, Rio de Janeiro 21941-909, Brazil

## Abstract

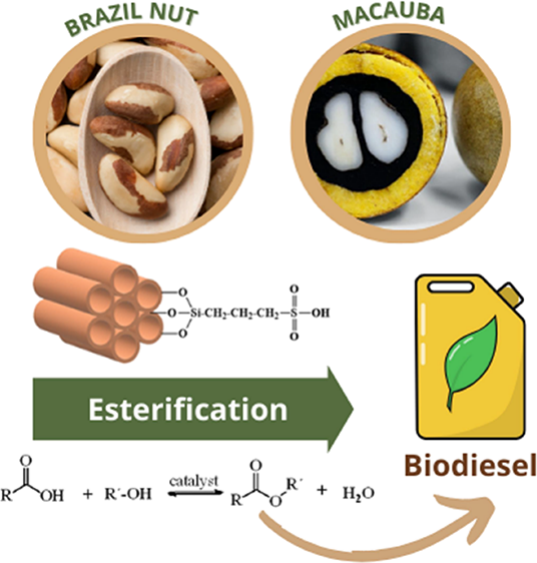

Low-value vegetable oils of Macauba pulp (*Acrocomia aculeata*) and Brazil nut (*Bertholletia excelsa*) were esterified with methanol under heterogeneous acid catalysis conditions. The characterization of the raw vegetable oils revealed high free fatty acid and moisture content. The heterogeneous acid catalysts were prepared by co-condensation upon the grafting of propylsulfonic acid on MCM-41 (Pr-HSO_3_/MCM-41) and SBA-15 (Pr-HSO_3_/SBA-15) mesoporous silica materials. The catalysts were used in the esterification of the Macauba (*Acrocomia aculeata*) and Brazil nut (*Bertholletia excelsa*) oils. The yield of methyl esters reached up to 96%. The catalysts were easily recovered and tested for reuse. The catalyst grafted on the MCM-41 silica showed faster deactivation than the catalyst grafted on SBA-15, due to more severe leaching of the active phase. The results obtained during the esterification of the Macauba and Brazil nut oils indicated that the catalysts could be promising materials for producing biodiesel from raw materials containing high-acidity values.

## 1 Introduction

Transesterification is the main route to produce biodiesel from triacylglycerides (TAG) of vegetable or animal origin using methanol or ethanol. The reaction is usually conducted in solution upon the use of sodium hydroxide or sodium methoxide, yielding methyl or ethyl esters of the fatty acids and glycerol.^1^ However, the purity of the TAG is crucial for base-catalyzed transesterification, requiring a low degree of moisture, phosphatides, and free fatty acids (FFA). These drawbacks are responsible for the increased process costs and the price of the vegetable oil accounts for more than 85% of the total costs of biodiesel production.^2^ Normally, the raw materials with high acid values (AI > 0.5%) and water content (H_2_O > 0.25%) are not suitable for basic transesterification because the water contributes to the hydrolysis of the esters, whereas the fatty acids neutralize the basic catalyst, which may also lead to soap formation, making the separation and purification more difficult.^3^

Low-value oleaginous materials, with high free fatty acid content, have become an attractive alternative to reduce process costs. They contain free fatty acids in the range of 10 to 35% and high moisture content, which affect the base-catalyzed transesterification route for biodiesel production. A viable alternative to allow the use of low-quality raw materials in biodiesel processing is the initial esterification to reduce the fatty acid content. In addition, the use of heterogeneous acid catalysts may facilitate the separation and permit reutilization, also contributing to cost reduction.^4,5^

Different heterogeneous catalysts based on resins, supported heteropolyacids, novel ionic liquid-supported composites, metal–organic frameworks, solid catalysts with magnetic structure, sulfated zirconia oxides, titanium oxides, or tungsten oxides^4−11^ have been used in the acid-catalyzed esterification and transesterification of low-quality raw materials. The main disadvantage is the range of temperature used, usually around 170–220 °C, to achieve satisfactory biodiesel yield from low-quality oil feedstocks. Another relevant problem is the leaching of active species, leading to deactivation of the solid catalysts.^10,11^

The mesostructured SBA-15 and MCM-41 materials have large porosity that assures excellent mass transfer properties and adjustment of the hydrophobic and hydrophilic properties through silylation.^9^ They can be functionalized with sulfonic acid groups and present good catalytic activity in the esterification and transesterification of vegetable oils with high acid values. The SBA-15 and MCM-41 mesoporous silica materials show high surface areas that allow the grafting of functional groups while minimizing diffusion limitations of reagents and products in the porous structure.^4,5,9^

There are several reports on the synthesis and application of acid and basic heterogeneous catalysts supported on mesoporous silicas, such as SBA-15 and MCM-41, aiming at producing biodiesel through esterification or transesterification of vegetable oils. The mesoporous silica may be functionalized with sulfonic acid or amines for the esterification-transesterification of vegetable native oils of high FFA content.^6−8^

Propylsulfonic acid (Pr-HSO_3_) and arene-sulfonic acid (Ar–HSO_3_) have been anchored on MCM-41 and SBA-15 mesoporous silicas to produce active catalysts for biodiesel production using poor-quality raw materials.^5^ They were used in the transesterification of the native *Jatropha curcas* oil with methanol. The functionalized SBA-15 showed higher catalytic activity and resistance to water and fatty acids than Amberlyst-36 and SAC-13 ion exchange resins.^12−14^

Brazil is one of the largest world producers of vegetable oils. Macauba (*Acrocomia aculeata*) is a palm tree native to the semiarid region and is found in a vast distribution area from Mexico to Argentina, also abundantly found in the Antilles. In Brazil, the Macauba tree is considered the most widely distributed palm tree. Another notable tree is the Brazil nut (*Bertholletia excelsa*), a large tree that is common in the northern region. Brazil nut oil is rich in unsaturated fatty acids and is widely used in the processing of pharmaceuticals and cosmetics. The oil can be an important feedstock for the production of biodiesel. The composition of Macauba and Brazil nut oils is shown in Table 1.^15,16^

**Table 1:** General Fatty Acid Composition of Macauba and Brazil Nut Oils

	**Macauba oil (%)**		
**fatty acid**	**pulp**	**almond**	Brazil nut (%)
caprylic (C8:0)	0.5	6.2	
capric (C10:0)	0.3	5.3	
lauric (C12:0)	2.0	43.6	
myristic (C14:0)	0.5	8.5	0.1
palmitic (C16:0)	18.7	5.3	18.0
palmitoleic (C16:1)	4.0	2.3	0.7
stearic (C18:0)	2.8	2.4	13.7
oleic (C18:1)	53.4	25.5	47.0
linoleic (C18:2)	17.7	3.3	
linolenic (C18:3)	1.50	1.92	15.0
others			5.6

Recently, we reported a sequential esterification and transesterification of Babassu and Pequi oils, of high acid content, using MCM-41 mesoporous silica functionalized with sulfonic groups and organic amines.^17^ There are few studies on biodiesel production from Brazil nut and Macauba oils using heterogeneous catalysts.^18−20^

In the present work, heterogeneous acid catalysts were prepared by co-condensation upon the grafting of propylsulfonic acid on MCM-41 (Pr-HSO_3_/MCM-41) and SBA-15 (Pr-HSO_3_/SBA-15) mesoporous silica materials and used in the esterification of the Macauba (*Acrocomia aculeata*) and Brazil nut (*Bertholletia excelsa*) oils of high acid content for biodiesel production.

## 2 Experimental Part

### 2.1 Materials

The native oils of Macauba pulp (*Acrocomia aculeata*) and Brazil nut (*Bertholletia excelsa*) were used as the raw material for esterification with methanol (Vetec, 99.8%, Brazil). Tetraethyl-orthosilicate (TEOS, Aldrich, 98%, Brazil) was used as a source of silica; hexadecyltrimethylammonium bromide (CTAB, Sigma, 99%, Brazil) and triblock copolymer Pluronic P123 (Aldrich, 95%, Brazil) were used as templates for the synthesis of MCM-41 and SBA-15 materials, respectively. (3-Mercaptopropyl)-trimethoxysilane (MPTMS) (Aldrich, 95%, Brazil) was used as the source of sulfonic acid, after oxidation with 30% hydrogen peroxide (Vetec, Brazil). The catalysts were synthesized using the procedures described in the literature.^21−24^

### 2.2 Synthesis of Pr-HSO_3_/SBA-15

The Pr-HSO_3_/SBA-15 was synthesized by co-condensation, which involves the synthesis of the inorganic porous support (MCM-41 and SBA-15), along with functionalization with the propylsulfonic acid moiety. In a typical synthesis, 4 g of P123 (0.069 mmol) was dissolved in 125 mL of 1.9 mol·L^–1^ HCl solution under vigorous stirring at room temperature for 30 min. The mixture was then heated to 40 °C until complete dissolution of the surfactant, which turned the solution clear. Then, 8 mL (36.9 mmol) of TEOS was added dropwise, and the white suspension was maintained under vigorous stirring at 40 °C for 1 h. Afterward, 0.76 mL of MPTMS (4.1 mmol) and 125 mL of H_2_O_2_ (39 mmol) were added to the medium. The mixture was kept under vigorous stirring at 40 °C for 20 h and hydrothermally aged at 110 °C for 24 h under static conditions. The suspension was filtered and the solid was dried overnight. The surfactant was removed by successive washings with boiling ethanol for 6 h. The white solid obtained was dried at 120 °C for 2 h.^21−23^

### 2.3 Synthesis of Pr-HSO_3_/MCM-41

A mixture with a molar composition of 27.5 mmol of CTAB (10 g), 89.0 mmol of ethanol (41 g), and 2.94 mol of deionized H_2_O (53 g) was used to synthesize the Pr-HSO_3_/MCM-41catalyst by co-condensation. The suspension was vigorously stirred at room temperature until complete dissolution of the surfactant. Then, 80.0 mmol of TEOS (18 mL) was added dropwise, followed by 20.0 mmol of MPTMS (3.1 mL). The mixture was aged for 24 h at room temperature with continuous stirring. The resulting product was separated by filtration and dried overnight. Successive washes with boiling ethanol extracted the surfactant over 6 h. The thiol groups were oxidized in a methanolic solution containing 2.04 g of H_2_O_2_ per gram of solid. The suspension was stirred at room temperature for 24 h and then washed with deionized water and ethanol. The material was filtered and acidified with 0.1 mol·L^–1^ solution of H_2_SO_4_ for 4 h. The material was filtered off again and dried at 120 °C for 2 h.^24^

### 2.4 Characterization of the Raw Materials

The acid value (AI), free fatty acid content (FFA), moisture content (H_2_O), and saponification value (sap) of the crude and processed vegetable oils were determined as described in the literature.^5,25^

The oils were divided into three representative samples. Three aliquots were taken from each sample, and triplicate assays were performed, totaling 72 measurements. The acidic value was determined by titration. In a typical experiment, 2.0 g of the sample was weighted into a 250 mL Erlenmeyer together with 25 mL of ethanol and 3–5 droplets of phenolphthalein as an indicator. The sample was titrated with standard 0.1 mol·L^–1^ NaOH solution until the turning point was reached. The acid value was expressed in mg_KOH_·g^–1^ of the sample and calculated by eq 1:
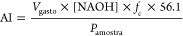
1where *V*_gasto_is the spent volume of NaOH in the titration, *f*_c_is the correction factor of NaOH solution, *P*_amostra_is the sample weight, [NaOH] is the molar concentration of NaOH, and 56.1 is the molar mass of KOH.

### 2.5 Determination of the Free Fatty Content

The free fatty acid content was determined by analogy to the acid value. The percentage of free fatty acids, in terms of oleic acid (OA), was calculated according to eq 2.
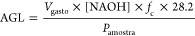
2where 28.2 corresponds to the milliequivalent gram of oleic acid.

### 2.6 Determination of the Moisture Content

The determination of the humidity was carried out by directly heating the oils at 105 °C. The content was determined gravimetrically by the mass difference between the wet and the dry samples, after the temperature treatment. Therefore, the samples were heated in an oven for 2 h at 105 ± 5 °C. The percentage of water contained in the samples was determined by eq 3.
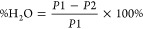
3where *P*1 is the wet sample weight and *P*2 is the dry sample weight.

### 2.7 Determination of the Saponification Value

This parameter was determined by the Koesttstafer method. A sample of the oils was mixed with a 4% alcoholic solution of KOH and kept at room temperature for 30 min, followed by titration with 0.5 mol·L^–1^ HCl solution. Similarly, a reference sample was titrated. The saponification value was then determined as shown in eq 4.
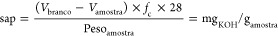
4where *V*_amostra_is the volume of HCl spent on titration of the sample, *V*_branco_ is the volume of HCl spent on titration of the reference sample, *f*_c_ is the correction factor for the molarity of the solution used for titration, and Peso_amostra_ is the sample weight.

### 2.8 Characterization of Catalysts

The elemental analysis in terms of carbon (C) and hydrogen (H) was determined at the Analytical Center of the University of São Paulo (USP). The sulfur content (S) was determined by inductively coupled plasma atomic emission spectroscopy (ICP-OES). Throughout the preparation of the samples, dry opening or deposition was performed by melting the sample with NaOH in a muffle at 280 °C (1 °C·min^–1^) for 2 h. The ash formed was solubilized in 0.01 mol·L^–1^ HCl solution, and the mixture was filtered and analyzed.

The textural properties were determined by N_2_ adsorption–desorption isotherms in a Micromeritics ASAP 2020 equipment. The specific surface area (*A*_BET_) was calculated by the Brunauer–Emmet–Teller (BET) method.^26^ The mean pore size distribution was derived from the adsorption–desorption isotherms of N_2_, which were measured at -196 °C, using the Barret–Joyner–Halenda method (BJH).

Fourier-transformed infrared (FTIR) spectra were recorded in the range 400–4000 cm^–1^ using a PerkinElmer model 100 spectrophotometer, with a spectral resolution of 4 cm^–1^. Solid-state nuclear magnetic resonance spectroscopy of ^29^Si (MAS NMR) was performed on a Bruker spectrometer, Avance III400 (9.4T), operating at Larmor frequencies of 79.51 MHz. The catalysts were also analyzed by thermogravimetry on a TA Instruments thermal analyzer, model SDT 2960, with a heating rate of 10 °C/min, in a N_2_ atmosphere (50 mL·min^–1^), in the range of 28–900 °C.

### 2.9 Catalytic Tests

The esterification was carried out in a stainless-steel Parr reactor. In a typical experiment, 2.5 g (9.0 mmol) of vegetable oil was added to 2.3 g of methanol (72.0 mmol) and 0.5 g of catalyst (20% relative to the oil mass), with an oil-to-methanol molar ratio of 1:8, respectively, and stirring around 700 rpm. The reaction time was set to 120 min and the temperature to 90 °C. The product was recovered after centrifugation at 2500 rpm for 20 min. The lipidic phase was heated to 100 °C to eliminate the residual water and methanol.

The catalytic performance was assessed as a function of the FFA content of crude oil (%AGL_bruto_) and esterified oil (%AGL_esterified_). The yield was calculated using eq 5.
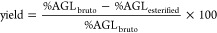
5

Control tests were carried out under the same experimental conditions without a catalyst and with pure MCM-41 and SBA-15 silica materials.

### 2.10 Reuse of the Catalysts

After separation by centrifugation, the heterogeneous acid catalysts were washed with methanol and *n*-hexane to remove polar and nonpolar compounds on the surface. Afterward, they were dried at 80 °C for 4 h. The recovered catalysts were reused in the esterification of vegetable oil under the same operating conditions described before.

### 2.11 Leaching Tests

Leaching is characterized by the loss of active sites upon dissolution in the reaction medium. The leaching test consisted of contacting 0.25 g of the catalyst with 2.3 g (72.0 mmol) of methanol at 120 °C for 180 min. The reaction mixture was filtered, and 1.5 g of the filtrate was mixed with 2.5 g (9.0 mmol) of the vegetable oil under the same conditions as described before to assess the conversion.

## 3 Results and Discussion

### 3.1 Characterization of Vegetable Oils

Table 2 shows the properties of the vegetable oils in their native forms. For the production of biodiesel, the FFA content should not exceed 0.5 wt %, whereas moisture cannot be above 0.25 wt %.^3^ One can see that the native Brazil nut and Macauba oils present values far above these threshold limits, which are not indicated for the production of biodiesel through base-catalyzed transesterification.

**Table 2:** Characterization of the Vegetable Oils

**samples**	**AI (mg KOH/g)**	**FFA (wt %)**	**H**_**2**_**O (wt %)**	**sap (mg KOH/g)**
Brazil nut oil (native)	5.38 (±0.02)	2.71 (±0.11)	0.33 (±0.4)	228
Macauba oil (native)	67.5 (±0.8)	34 (±0.3)	0.8 (±0.03)	235

The saponification value (sap) is the number of milligrams of potassium hydroxide required to neutralize the free fatty acids resulting from the hydrolysis of one gram of the sample.

### 3.2 Characterization of the Acid Catalysts

#### 3.2.1 Elemental Analysis

**Figure 1:**
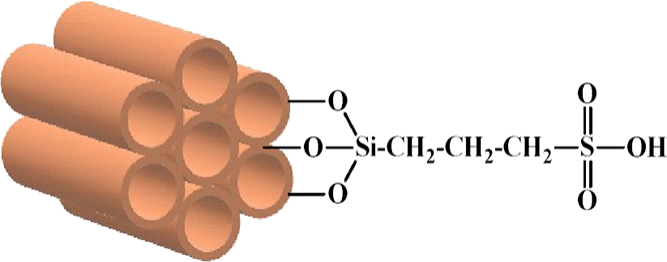
Molecular structure of the functionalized acid catalysts.

The degree of functionalization of propylsulfonic acid on the MCM-41 and SBA-15 mesoporous silicas was determined by elemental analysis. Table 3 shows the CHS results and the amount of acid sites (*N*_CSF_) in terms of mmol g^–1^ of catalyst obtained by Bohem titration.^27^ The results revealed significant functionalization with acid sites on both catalysts (Figure 1). The Pr-HSO_3_/MCM-41 showed an acidity of 287 mmol g^–1^ and Pr-HSO_3_/SBA-15 exhibited 282 mmol g^–1^ acid sites. These results show that both mesoporous silica supports have approximately the same acidity, although the amount of sulfur was higher in the SBA-15 material. This apparent discrepancy may be explained by the incomplete oxidation of the sulfur atoms or by the incorporation of sulfur at the micropores of the SBA-15 structure.

**Table 3:** Elemental Analysis and Acidity of the Heterogeneous Acid Catalysts^a^

catalyst	C (%)	H (%)	S (%)	*N*_CSF_ (mmol g^–1^ of catalyst)
Pr-HSO_3_/MCM-41	2.5	4.0	1.3	287
Pr-HSO_3_/SBA-15	8.0	7.0	2.4	282

a Legend: N_CSF_, number of acid sites on the surface of the catalyst.

#### 3.2.2 Textural Properties

The BET area and pore diameter are shown in Table 4. For comparison purposes, the area of pure SBA-15 and MCM-41 mesoporous silica was shown and was similar to those described in the literature.^28,29^ The SBA-15 has thick walls, offering a robust material with good thermal and chemical stability. It is a mesoporous material with hexagonally ordered pores, which may range from 0.5 to 6.0 nm. On the other hand, the MCM-41 material consists of hexagonally ordered pores, with surface areas of up to 1200 m^2^.g^–1^. Besides that, MCM-41 exhibits a large pore volume and thin walls. When subjected to microscopy, they reveal one-dimensional pores similar to a honeycomb.

**Table 4:** Textural Properties of the Functionalized Acid Catalysts^a^

	textural properties		
catalyst	***S***_**BET**_**(m**^**2**^**·g**^**–1**^**)**	***V***_**p**_**,**_**BJH**_**(cm**^**3**^**·g**^**–1**^**)**	***D*_pBJH_(nm)**
SBA-15	630	0.460	5.2
Pr-HSO_3_/SBA-15	14	0.014	3.42
MCM-41	1134	1.020	2.32
Pr-HSO_3_/MCM-41	63	0.074	4.27

a *S*_BET_ = surface area; *V*_p,BJH_ = pore volume; *D*_p,BJH_ = average pore diameter.

Upon functionalization, the pore volume, surface area, and pore diameter of the supports are decreased. This is consistent with the presence of the organic moiety, which may fill the pores. Nevertheless, the textural properties are consistent with the results of the literature.^30−33^

#### 3.2.3 Fourier-Transform Infrared Spectroscopy (FTIR)

**Figure 2:**
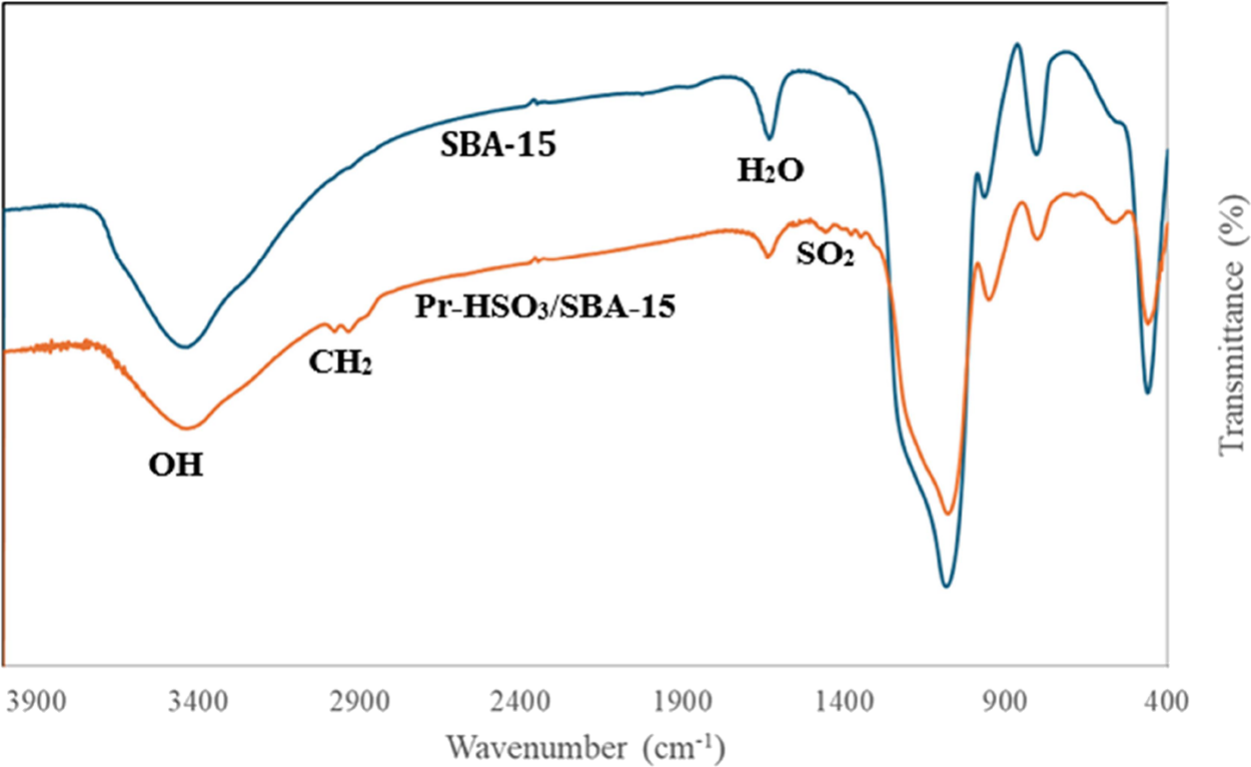
FTIR spectra of SBA-15 mesoporous silica and Pr- HSO_3_/SBA-15.

**Figure 3:**
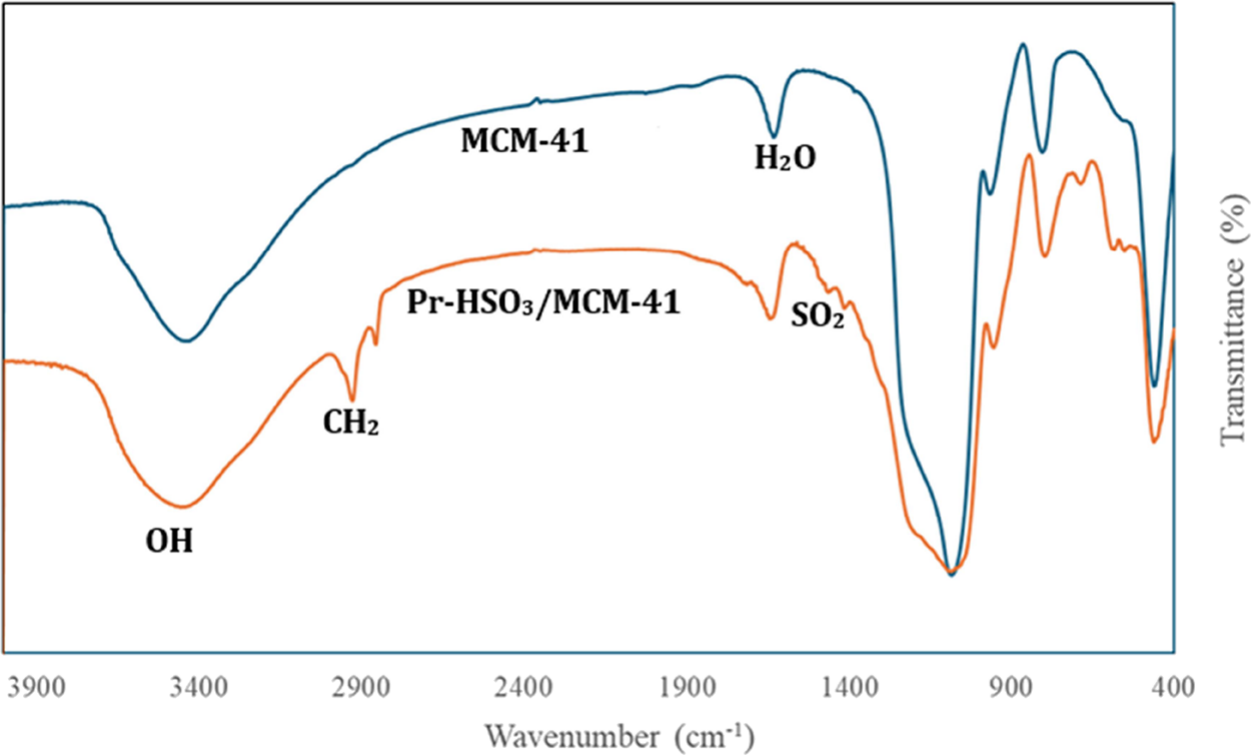
FTIR-spectra of MCM-41 mesoporous silica and the Pr-HSO_3_/MCM-41.

The FTIR spectroscopy was conducted to verify the anchoring of the sulfonic acid groups on the surface of the catalysts. Figures 2 and 3 show the FTIR spectra of the mesoporous supports and the acid catalysts Pr-HSO_3_/SBA-15 and Pr-HSO_3_/MCM-41, respectively. All of the mesoporous silica materials showed absorption bands in the range of 1644–1632 cm^–1^, suggestive of water on the surface of the materials. The condensed broad absorption bands between 3452 and 3420 cm^–1^ were attributed to the OH group on the surface, as well as adsorbed water.

The functionalized Pr-HSO_3_/MCM-41 and Pr-HSO_3_/SBA-15 catalysts presented absorption bands between 1463 and 1377 cm^–1^, due to the asymmetric stretching (ν_as_) of the SO_2_-group, as well as bands in the region of 1725 cm^–1^ and between 2934 and 2932 cm^–1^, indicating the asymmetric (ν_as_) and symmetric (ν_s_) stretching of the CH_2_ bonds of the propyl chain. The presence of these bands indicates successful functionalization of the mesoporous silica with the sulfonic acid moiety.^34−36^

#### 3.2.5 Solid-State NMR Spectroscopy

**Figure 4:**
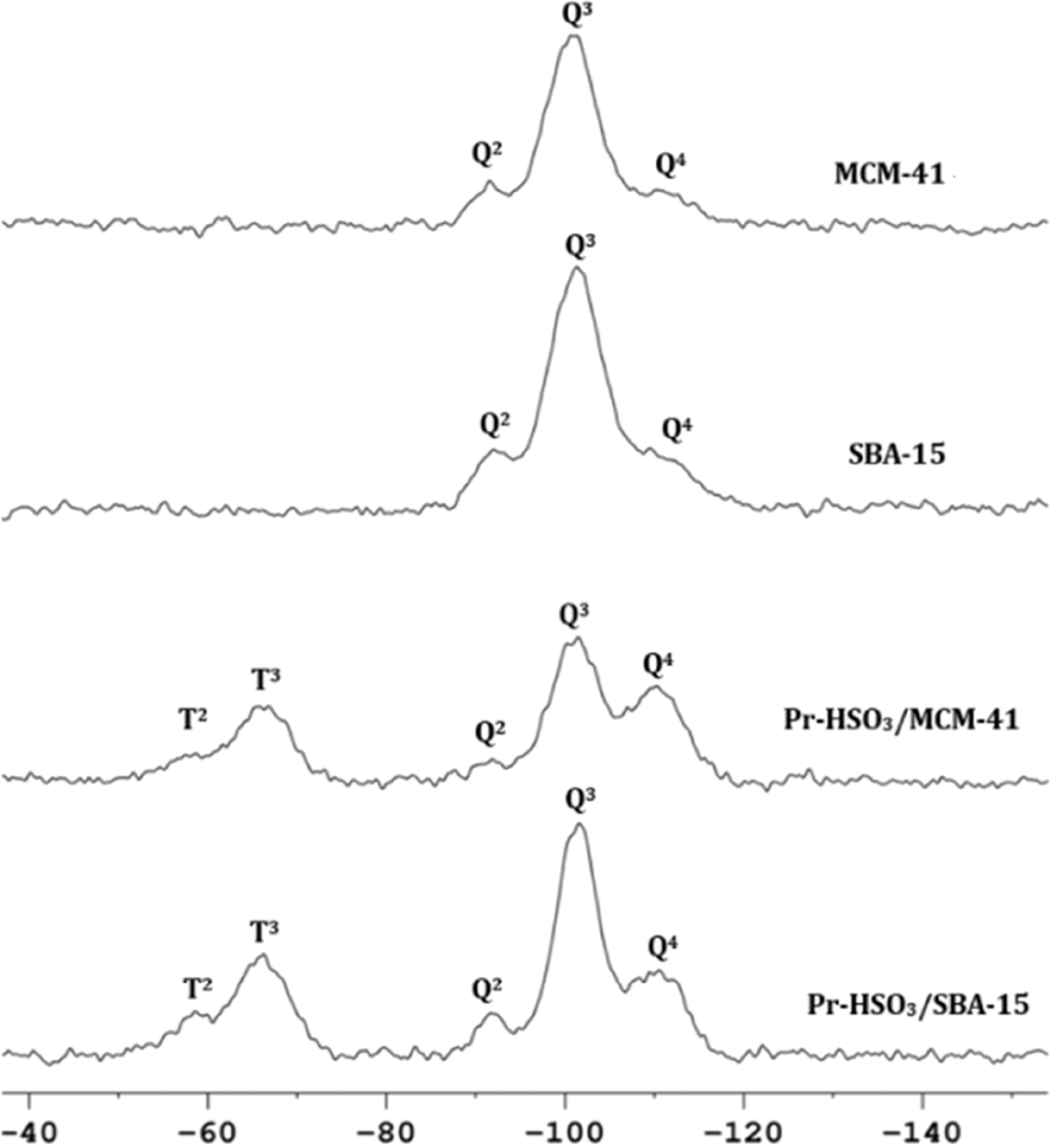
^29^Si MAS/NMR spectra of the mesoporous silicas and functionalized acid catalysts.

The ^29^Si MAS/NMR provides information regarding the grafting of the organic moiety on the mesoporous silica. Figure 4 illustrates the ^29^Si MAS/NMR spectra of mesoporous silica and functionalized catalysts. The pure MCM-41 silica showed Q^2^ (−91 ppm), Q^3^ (−101 ppm), and Q^4^ type (−111 ppm) sites, with the Q^3^ site [Si(OSi)_3_OH] being more intense. A similar behavior was observed for the SBA-15 material, showing the Q^2^ (−92 ppm), Q^3^ (−101 ppm), and Q^4^ (−112 ppm) peak.^35,36^

When comparing the spectra of the Pr-HSO_3_/MCM-41 catalyst with pure MCM-41, a small displacement of the Q^2^ and Q^3^ peaks. In the spectra of the functionalized materials, besides the Q,^2^ Q,^3^ and Q^4^ peaks, there appear the T^2^ (−59 ppm) and T^3^ (−67 ppm) peaks associated with C–Si bonds, which confirms the functionalization of the silicas.^35,36^

#### 3.2.6 Thermogravimetric Analysis

**Figure 5:**
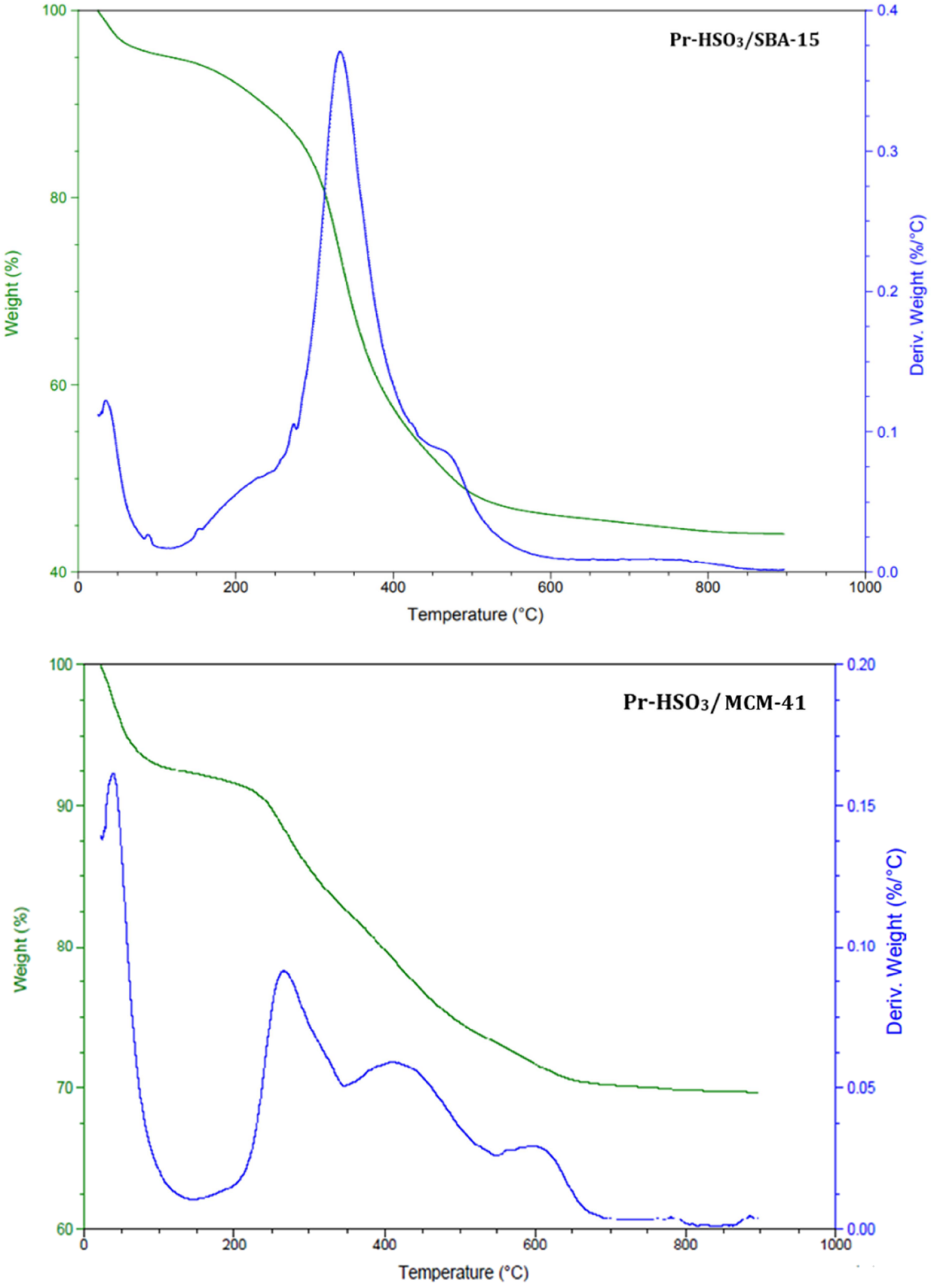
Thermogravimetric curves of the functionalized acid catalysts.

Figure 5 shows the thermogravimetric (TG) curves and the first thermogravimetric derivative (DTG) of the catalysts. The Pr-HSO_3_/SBA-15 catalyst showed three events of mass loss. The first occurred from room temperature to 120 °C, with 7.1% mass loss, and may be associated with water desorption. The second event goes from 120 to 575 °C, with 22.3% mass loss, and may be ascribed to the decomposition of the organic moiety. The third event occurs above 575 °C with approximately 0.8% mass loss and may be associated with loss of structural water and degradation of the silica support.^37^

The Pr-HSO_3_/MCM-41 catalyst showed a similar pattern, with the first event of water desorption taking place from room temperature up to 120 °C with a mass loss of 4.8%. The second and third events occur from 120 to around 650 °C, with a total mass loss of 48.7%.^38^

### 3.3 Catalytic Activity

The catalytic performance of the mesostructured silica functionalized with propyl sulfonic acid was evaluated in the esterification of the native vegetable oils of high free fatty acids content. Initially, control experiments were carried out without a catalyst and with pure silica supports. Table 5 shows the results.

**Table 5:** Esterification of Macauba and Brazil Nut Native Oils at 90 °C, 120 min, and Oil/Methanol Ratio of 1:8

	**FFA content (wt %)**		
native oils	**native**	**treated**^a^	yield (%)
Macauba oil			
No catalyst	34.0	27.0	20
MCM-41	34.0	11.0	68
Pr-HSO_3_/MCM-41	34.0	2.0	94
SBA-15	34.0	22.0	35
Pr-HSO_3_/SBA-15	34.0	6.1	82
Brazil nut oil			
No catalyst	13.0	6.0	54
MCM-41	13.0	7.0	46
Pr-HSO_3_/MCM-41	13.0	0.5	94
SBA-15	13.0	3.0	23
Pr-HSO_3_/SBA-15	13.0	0.7	97

a After reaction with methanol at 90 °C and 120 min.

One can see that the pure mesoporous silica supports have significantly lower catalytic activity than the functionalized materials and slightly higher than the noncatalyzed reaction when the Macauba oil is considered. For the Brazil nut oil, the results with the pure silica materials were slightly worse than those without the catalyst. The interpretation may reside in the lower amount of FFA in this oil. Hence, the adsorption of the FFA on the supports reduces the acidity of the medium and, consequently, reduces the self-esterification compared with the uncatalyzed reaction.

For the functionalized materials, Pr-SO_3_/MCM-41 showed better catalytic performance, especially for the Macauba oil, which has a larger FFA content. Since the acidities of both functionalized catalysts are mostly the same, the difference may be associated with the higher BET area of the Pr-SO_3_/MCM-41 catalyst, allowing better diffusion and access of the reactants to the acid sites. This is particularly important for oils with a high FFA content.

The Pr-HSO_3_/SBA-15 and Pr-HSO_3_/MCM-41 catalysts were subjected to leaching tests; the results showed the formation of 0.2 and 6% of methyl esters, respectively, indicating that leaching is negligible on the Pr-HSO_3_/SBA-15 catalyst and of low to moderate significance on the Pr-HSO_3_/MCM-41 catalyst.

Several studies in the literature report the use of solid acid catalysts with low-value feedstocks because these catalysts can also catalyze the transesterification of the oil along with the esterification of FFAs in a single process, which could eventually reduce the costs of biodiesel production.^39,40^ Nevertheless, the temperatures used for this kind of process are usually higher than the temperature used in the present experiments, and therefore, we do not believe that transesterification is significant in these cases.

**Figure 6:**
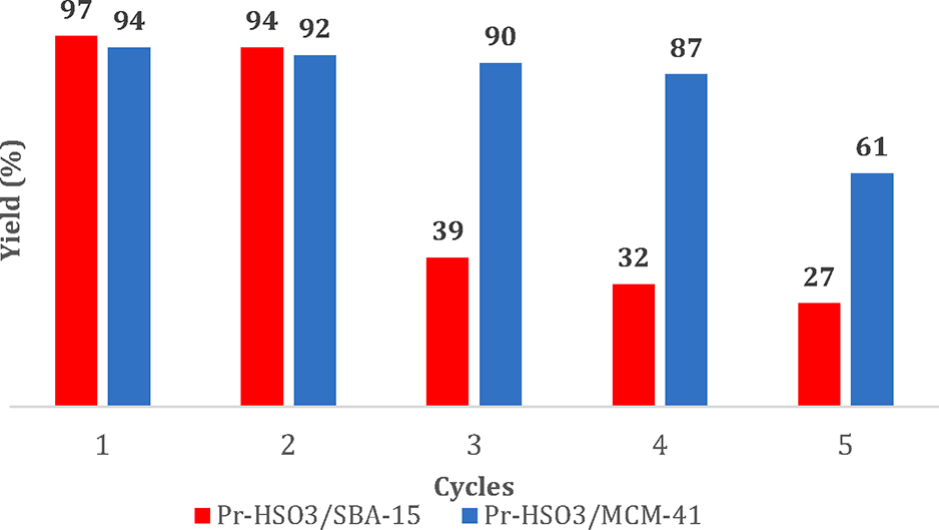
Reusability of the functionalized acid catalysts.

Figure 6 shows the reuse of the functionalized catalysts. One can see that Pr-HSO_3_/MCM-41 significantly decreases the yield of methyl esters upon the third consecutive use. This may be explained by the leaching of the active phase, as mentioned before. On the other hand, the Pr-HSO_3_/SBA-15 keeps almost the same activity during four consecutive runs, still maintaining a reasonable yield of methyl ester in the fifth run. These results may also be interpreted in terms of the partial leaching of the active phase, which was low for this catalyst but is observed to some extent. Alternatively, deactivation may be possible due to the progressive poisoning of the acid sites by adsorbed impurities of the oil. Anyway, the functionalization of the SBA-15 structure gives a more stable catalyst that can be reused at least for four consecutive runs without appreciable loss of activity. For further reutilization, the catalyst loading may be increased to compensate for the drop in activity.

Catalyst deactivation has been commonly reported in the literature upon reuse. The leaching of the active sites and the poisoning of the acid sites by adsorbed impurities of the oil have been mentioned as the most common causes of deactivation.^19,41^ The purpose of this work was not to go further into the deactivation mechanism of the catalysts. Nevertheless, we believe that the poisoning of the acid site is the main cause of the deactivation of the Pr-HSO_3_/SBA-15 catalyst, whereas leaching plays an important role in the deactivation of the Pr-HSO_3_/MCM-41 material.

The influence of the temperature and time was evaluated in the esterification of Brazil nut oil. The results are shown in Tables 6 and 7. It can be seen that increasing the temperature from 90 to 120 °C and prolonging the time from 120 to 180 min did not show a significant variation in the yield of the methyl ester for both catalysts. The results indicated that equilibrium has been reached after 120 min of reaction and that increasing the temperature slightly decreases the yield of the esters, probably because of a shift in the equilibrium of the reaction. The results of the esterification of crude Brazil Nut oil with the Pr-HSO_3_/SBA-15 catalyst were superior to the results described in the literature for the esterification of oils of high fatty acid content, indicating the potential of this catalyst for the production of biodiesel when raw materials of low quality are concerned.^42,43^

**Table 6:** Yield of Esterification of Brazil Nut Oil Using Pr-HSO_3_/SBA-15

esterification of native Brazil nut oil (Bertholletia excelsa)							
	amount of matter					reaction time (min)	
catalyst	oil (mol)	MeOH (mol)	catalyst (mmol/g)	FFA (%)	temp. (°C)	120	180
pr-HSO_3_/SBA-15	9 × 10^–3^	0.07	72	12	90	95	95
					120	92	89

**Table 7:** Yield of the Esterification of Brazil Nut Oil Using Pr-HSO_3_/MCM-41

esterification of native Brazil nut oil (*Bertholletia excelsa*)							
	amount of matter					reaction time (min)	
catalyst	oil (mol)	MeOH (mol)	catalyst (mmol/g)	FFA (%)	temp. (°C)	120	180
pr-HSO_3_/MCM-41	9 × 10^–3^	0.07	72	12	90	96	92
					120	94	91

## 4 Conclusions

The synthesis of heterogeneous acid catalysts upon the grafting of propyl sulfonic acid on MCM-41 and SBA-15 mesoporous silicas was successful. The functionalized catalysts presented basically the same acidity, although the sulfur content on the SBA-15 support was higher than that on the MCM-41. The discrepancy may be explained by the partial oxidation of the sulfur atoms or by the incorporation of functionalization at the micropores.

The catalysts presented high activity, up to 96% yield of methyl esters, during the esterification of crude oils of high acidity from Macauba pulp and Brazil nut. The Pr-HSO_3_/MCM-41 catalyst showed low to moderate leaching of the active phase and presented a significant decrease in activity after three consecutive runs. On the other hand, the Pr-SBA-15/SBA-15 catalyst showed low leaching and the catalytic activity could be maintained during four consecutive runs, with a more significant drop after the fifth reuse. These results pointed out that the functionalized catalysts have the potential to be used in the production of biodiesel from low-value raw materials, especially when the FFA content and moisture are above the limits for the use of basic transesterification.

## References

[ref1] Rezende M. J. C.; de Lima A. L.; Silva B. V.; Mota C. J. A.; Costa K. P.; Torres E. A.; da Rocha G. O.; Cardozo I. M. M.; Guarieiro L. L. N.; Pereira P. A. P.; Martinez S.; de Andrade J. B. Biodiesel: An Overview II. J. Braz. Chem. Soc. 2021, 32, 1301–1344. 10.21577/0103-5053.20210046.

[ref2] Alegría A.; de Arriba Á. L. F.; Morán J. R.; Cuellar J. Biodiesel production using 4-dodecylbenzenesulfonic acid as catalyst. Appl. Catal., B 2014, 160–161, 743–756. 10.1016/j.apcatb.2014.06.033.

[ref3] de Lima A. L.; Ronconi C. M.; Mota C. J. A. Heterogeneous Basic Catalysts for Biodiesel Production. Catal. Sci. Technol. 2016, 6, 2877–2891. 10.1039/C5CY01989C.

[ref4] Ezekannagha C. B.; Onukwuli O. D.; Nnanwube I. A.; Ezeamaku U. L.; Ohaegbulam C. M. Green hetero-alkali catalyst in optimized waste lard oil transesterification for biodiesel synthesis. Results in Chem. 2024, 11, 101797 10.1016/j.rechem.2024.101797.

[ref5] Melero J. A.; Bautista L. F.; Morales G.; Iglesias J.; Sánchez-Vázquez R. Biodiesel production from crude palm oil using sulfonic acid-modified mesostructured catalysts. Chem. Eng. J. 2010, 161, 323–331. 10.1016/j.cej.2009.12.037.

[ref6] de Rezende S. M.; de Castro Reis M.; Reid M. G.; Silva P. L. Jr.; Coutinho F. M. B.; da Silva San Gil R. A.; Lachter E. R. Transesterification of vegetable oils promoted by poly(styrene-divinylbenzene) and poly(divinylbenzene). Appl. Catal., A 2008, 349, 198–203. 10.1016/j.apcata.2008.07.030.

[ref7] Reis M. C.; Freitas F. A.; Lachter E. R.; San Gil R. A. S.; Nascimento R. S. V.; Poubel R. L.; Borré L. B. Biodiesel Production From Fatty Acids Of Refined Vegetable Oils By Heterogeneous Acid Catalysis And Microwave Irradiation. Quim. Nova 2015, 38, 1307–1312. 10.5935/0100-4042.20150163.

[ref8] Mulyatun M.; Prameswari J.; Istadi I.; Widayat W. Production of non-food feedstock-based biodiesel using acid-base bifunctional heterogeneous catalysts: A review. Fuel 2022, 314, 122749 10.1016/j.fuel.2021.122749.

[ref9] Ismaeel H. K.; Albayati T. A.; Al-Sudani F. T.; Salih I. K.; Dhahad H. A.; Saady N. M. C.; Zendehboudi S.; Fattah I. M. R. The role of catalysts in biodiesel production as green energy applications: A review of developments and prospects. Chem. Eng. Res. Des. 2024, 204, 636–653. 10.1016/j.cherd.2024.02.048.

[ref10] Xie W.; Wan F. Immobilization of polyoxometalate-based sulfonated ionic liquids on UiO66–2COOH metal-organic frameworks for biodiesel production via one-pot transesterification-esterification of acidic vegetable oils. Chem. Eng. J. 2019, 365, 40–50. 10.1016/j.cej.2019.02.016.

[ref11] Xie W.; Wang H. Immobilized polymeric sulfonated ionic liquid on core-shell structured Fe_3_O_4_/SiO_2_ composites: A magnetically recyclable catalyst for simultaneous transesterification and esterifications of low-cost oils to biodiesel. Renewable Energy 2020, 145, 1709–1719. 10.1016/j.renene.2019.07.092.

[ref12] de Lima A. L.; Mbengue A.; San Gil R. A. S.; Ronconi C. M.; Mota C. J. A. Synthesis of amine-functionalized mesoporous silica basic catalysts for biodiesel production. Catal. Today 2014, 226, 210–216. 10.1016/j.cattod.2014.01.017.

[ref13] Melero J. A.; van Grieken R.; Morales G. Advances in the synthesis and catalytic application of organosulfonic funcionalized mesoestructured materials. Chem. Rev. 2006, 106, 3790–3812. 10.1021/cr050994h. 16967921

[ref14] Mbaraka I. K.; Radu R. D.; Lin V. S-Y.; Shanks B. H. Organosulfonic acid funcionalized mesoporous silicas for the esterification of fatty acid. J. Catal. 2003, 219, 329–336. 10.1016/S0021-9517(03)00193-3.

[ref15] Lima C. R. R. C.; López-García P.; Tavares V. F.; Almeida M. M.; Zanolini C.; Aurora-Prado M. S.; Santoro M. I. R. M.; Kedor-Hackamnn E. R. M. Separation and identification of fatty acids in cosmetic formulations containing Brazil nut oil by capillary electrophoresis. J. Basic Appl. Pharm. Sci. 2011, 32, 341–348.

[ref16] Carvalho A. K. F.; Da Rós P. C. M.; Teixeira L. F.; Andrade G. S. S.; Zanin G. M.; de Castro H. F. Assessing the potencial of non-edible oils and residual fat to be used as a feedstock source in enzymatic ethanolysis reaction. Ind. Crops Prod. 2013, 50, 485–493. 10.1016/j.indcrop.2013.07.040.

[ref17] Moreira A. F. S. A.; Vieira J. S. C.; de Lima A. L.; Lachter E. R.; Mota C. J. A. Sequential Acid-Catalyzed Esterification and Base-Catalyzed Transesterification of Babassu (Attalea speciosa Mart. Ex Spreng.) and Pequi (Caryocar brasiliense Camb.) Oils of High Acid Values Over Functionalized Mesoporous Silicas. Waste Biomass Valorization 2024, 16, 1189 10.1007/s12649-024-02698-8.

[ref18] Visioli L. J.; de Castilhos F.; da Silva C. Use of heterogeneous acid catalyst combined with pressurized conditions for esters production from macauba pulp oil and methyl acetate. J. Supercrit. Fluids 2019, 150, 65–74. 10.1016/j.supflu.2019.03.023.

[ref19] Rodrigues K. L. T.; Pasa V. M. D.; Cren E. C. Kinetic modeling of catalytic esterification of non-edible macauba pulp oil using macroporous cation exchange resin. J. Environm. Chem. Eng. 2018, 6, 4531–4537. 10.1016/j.jece.2018.06.037.

[ref20] de Souza Gonçalves Proença B.; Fioroto P. O.; Heck S. C.; Duarte V. A.; Cardozo Filho L.; Feihrmann A. C.; Beneti S. C. Obtention of methyl esters from macauba oil using eggshell catalyst. Chem. Eng. Res. Des. 2021, 169, 288–296. 10.1016/j.cherd.2021.03.015.

[ref21] Melero J. A.; Bautista L. F.; Morales G.; Iglesias J.; Briones D. Biodisel production with heterogeneous sulfonic acid-functionalized mesostructured catalyst. Energy Fuels 2009, 23, 539–547. 10.1021/ef8005756.

[ref22] Ziarani G. M.; Lashgari N.; Badiei A. Sulfonic acid-funcionalized mesoporous sílica SBA-pr-SO_3_Has solid acid catalyst in organic reactions. J. Mol. Catal. A: Chem. 2015, 397, 166–191. 10.1016/j.molcata.2014.10.009.

[ref23] Margolese D.; Melero J. A.; Christiansen S. C.; Chmelka B. F.; Stucky G. D. Direct synthesis of ordered SBA-15 mesoporous containing sulfonic acid groups. Chem. Mater. 2000, 12, 2448–2459. 10.1021/cm0010304.

[ref24] Shang F.; Sun J.; Wu S.; Yang Y.; Kan Q.; Guan J. Direct synthesis of acid–base bifunctional mesoporous MCM-41 silica and its catalytic reactivity in Deacetalization–Knoevenagel reactions. Microporous Mesoporous Mater. 2010, 134, 44–50. 10.1016/j.micromeso.2010.05.005.

[ref25] Vieira J. S. C.; Sousa T. L.; Rosas L. S.; Lima A. L.; Ronconi C. M.; Mota C. J. A. Homogeneous esterification and transesterification of vegetable oils containing high free fatty acids. Quim. Nova 2017, 41, 10–16. 10.21577/0100-4042.20170148.

[ref26] Brunauer S.; Emmett P. H.; Teller E. Adsorption of gases in multimolecular layers. J. Am. Chem. Soc. 1938, 60, 309 10.1021/ja01269a023.

[ref27] Corro G.; Bañuelos F.; Vidal E.; Cebada S. Measurements of surface acidity from solid catalysts to free fatty acids esterification in crude jatropha for the production of biodiesel. Fuel 2014, 115, 625–628. 10.1016/j.fuel.2013.07.060.

[ref28] Beck J. S.; Vartuli J. C.; Roth W. J.; Leonowicz M. E.; Kresge C. T.; Schmitt K. D.; Chu C. T. W.; Olson D. H.; Sheppard E. W.; McCullen S. B.; Higgins J. B.; Schlenker J. L. A new family of mesoporous molecular sieves prepared with liquid crystal templates. J. Am. Chem. Soc. 1992, 114, 10834–10843. 10.1021/ja00053a020.

[ref29] Meynen V.; Cool P.; Vansant E. F. Verified syntheses of mesoporous materials. Microp. Mesop. Mater. 2009, 125, 170–223. 10.1016/j.micromeso.2009.03.046.

[ref30] Wu P.; Tatsumi T.; Komatsu T.; Yashima T. Postsynthesis, Characterization, and Catalytic Properties in Alkene Epoxidation of Hydrothermally Stable Mesoporous Ti-SBA-15. Chem. Mater. 2002, 14, 1657–1664. 10.1021/cm010910v.

[ref31] Xie W.; Yang X.; Fan M. Novel solid base catalyst for biodiesel production: mesoporous SBA-15 silica immobilized with 1–3-dicyclohexyl-2-octylguanidine. Renew. Energy 2015, 80, 230–237. 10.1016/j.renene.2015.02.014.

[ref32] Cai W.; Yu J.; Jaroniec M. Effect of nonionic structure-directing agents on adsorption and structural properties of mesoporous alumina. J. Mater. Chem. 2011, 21, 9066–9072. 10.1039/c1jm10642b.

[ref33] Jeenpadiphat S.; Bjork E. M.; Oden M.; Tungasmita D. N. Propylsulfonic acid functionalized mesoporous silica catalysts for esterification of fatty acids. J. Mol. Catal. A 2015, 410, 253–259. 10.1016/j.molcata.2015.10.002.

[ref34] Zhang P.; Wu H.; Fan M.; Sun W.; Jiang P.; Dong Y. Direct and postsynthesis of tin-incorporated SBA-15 functionalized with sulfonic acid for efficient biodiesel production. Fuel 2019, 235, 426–432. 10.1016/j.fuel.2018.08.029.

[ref35] Han O. H.; Baea Y. K. Solid-state NMR study on the structure and dynamics of triblock copolymer p123 remaining in SBA-15 after solvent washing. Bull. Korean Chem. Soc. 2008, 29, 911 10.5012/bkcs.2008.29.5.911.

[ref36] Lima A. L.; Vieira J. S. C.; Ronconi C. M.; Mota C. J. A. Tailored hybrid materials for biodiesel production: Tunning the base type, support and preparation method for the best catalytic performance. Mol. Catal. 2018, 458, 240–246. 10.1016/j.mcat.2017.09.032.

[ref37] Usai E. M.; Sini M. F.; Meloni D.; Solinas V.; Salis A. Sulfonic acid-functionalized mesoporous silicas: Microcalorimetric characterization and catalytic performance toward biodiesel synthesis. Microporous Mesoporous Mater. 2013, 179, 54–62. 10.1016/j.micromeso.2013.05.008.

[ref38] Díaz I.; Mohino F.; Pérez-Pariente J.; Sastre E. Synthesis, characterization and catalytic activity of MCM-41-type mesoporous silicas functionalized with sulfonic acid. Appl. Catal., A 2001, 205, 19–30. 10.1016/S0926-860X(00)00808-5.

[ref39] Zhang G.; Xie W. Hierarchical porous SAPO-34 decorated with Mo and W oxides for concurrent transesterification-esterifications for efficient biodiesel production from acidic soybean oil. Renew. Energy 2024, 222, 119927 10.1016/j.renene.2023.119927.

[ref40] Hou S.; Xie W. Three-dimensional hierarchical meso/macroporous Mo/Ce/TiO_2_ composites enhances biodiesel production from acidic soybean oil by transesterification-esterifiications. Energy Conv. Manag. 2024, 305, 118273 10.1016/j.enconman.2024.118273.

[ref41] Naseef H. H.; Tulaimat R. H. Transesterification and esterification for biodiesel production: A comprehensive review of catalysts and palm oil feedstocks. Energy Convers. Manag. 2025, 26, 100931 10.1016/j.ecmx.2025.100931.

[ref42] Zuo D.; Lane J.; Culy D.; Schultz M.; Pullar A.; Waxman M. Sulfonic acid functionalized mesoporous SBA-15 catalysts for biodiesel production. Appl. Catal., B 2013, 129, 342–350. 10.1016/j.apcatb.2012.09.029.

[ref43] Shah K. A.; Parikh J. K.; Maheria K. C. Use of sulfonic acid-functionalized silica as catalyst for esterification of free fatty acids (FFA) in acid oil for biodiesel production: an optimization study. Res. Chem. Intermed. 2015, 41, 1035–1051. 10.1007/s11164-013-1253-6.

